# Dr. F. S. Kaynor

**Published:** 1917-02

**Authors:** 


### OBITUARY

Readers are requested to report promptly the death of all physicians in Western New York, or former residents of this region, or graduates of any medical school in Western New York, and to notify the families of the deceased of our desire to publish adequate Obituary notices.

---

**Dr. F. S. Kaynor,** Geneva (class not stated) died at his home in Amea, Ia., Dec. 22, of paralysis, aged 95. He was about to celebrate his 71st wedding anniversary. He formerly practiced medicine in N. Y., Wis., Mo. and finally at Cedar Rapids, Ia., but retired from active practice some years ago. His wife survives him.

